# Tissue extracellular vesicles suppress neonatal cardiac regeneration: a Pak2-Erk1/2-mediated macrophage paracrine signaling: P8-EVs suppress neonatal cardiac regeneration

**DOI:** 10.3724/abbs.2024193

**Published:** 2025-02-14

**Authors:** Yongwei Li, Laihai Zhang, Yating Wu, Lu Wei, Zhenchun Zhang, Hanling Mo, Zhongmin Liu, Xianyun Wang, Yunli Shen, Hongming Zhu

**Affiliations:** 1 Institute for Regenerative Medicine & Research Center for Translational Medicine Shanghai East Hospital School of Medicine Tongji University Shanghai 200092 China; 2 Department of Cardiology Shanghai East Hospital School of Medicine Tongji University Shanghai 200092 China; 3 Department of Cardiovascular Surgery Shanghai East Hospital School of Medicine Tongji University Shanghai 200092 China; 4 Key Laboratory of Microbial Drugs Innovation and Transformation of Yan′an School of Basic Medicine Yan’an University Yan’an 716000 China; 5 Department of Cardiology Shanghai East Hospital of Clinical Medical College Nanjing Medical University Nanjing 211166 China; 6 Heart Center the First Hospital of Hebei Medical University Shijiazhuang 050031 China; 7 Hebei Provincial Key Laboratory of Cardiac Injury Repair Mechanism Study Shijiazhuang 050031 China

**Keywords:** myocardial infarction (MI), extracellular vesicle (EV), cell proliferation

## Abstract

Myocardial infarction leads to cardiomyocyte loss, and the compromised proliferative capacity of cardiomyocytes after birth hinders the process of heart repair, ultimately culminating in heart failure. Extracellular vesicles (EVs), known as cell-secreted “messengers”, play a pivotal role in tissue pathophysiology. Here, we report the novel finding that myocardial tissue-derived vesicles from mice on postnatal day 8 (P8-EVs) possess the potential to modulate cardiomyocyte proliferation. Notably, direct administration of EVs derived from day 1 or day 8 (P1/P8) myocardial tissue does not impact neonatal cardiomyocyte proliferation or myocardial repair in mice with myocardial infarction. However, by leveraging bioinformatics, high-throughput omics, and single-cell analyses, we unveil that P8-EVs are enriched with the key gene p21 activated kinase 2 (Pak2), a regulator of macrophage reparative function. Through single-cell sequencing of P8 myocardial tissue, we identify macrophages as the cell type with the highest Pak2 content, implying a close association between macrophages and P8-EV function. Intriguingly, further investigations reveal that P8-EVs significantly promote M1-like polarization, augment phagocytosis, and affect factor secretion in macrophages. Co-culture experiments demonstrate that P8-EV-treated macrophages strongly suppress neonatal cardiomyocyte proliferation, and this effect is effectively reversed by a Pak2 inhibitor. Additional pathway intervention experiments reveal that P8-EVs activate the downstream Erk1/2 signaling pathway of Pak2. Collectively, our findings indicate that P8-EVs regulate macrophage paracrine activities through the Pak2-Erk1/2 axis, thereby influencing cardiomyocyte proliferation. This finding reveals a potential underlying mechanism for the compromised proliferative capacity of cardiomyocytes in adult mice.

## Introduction

The regenerative capacity of the adult mammalian heart is limited, as it displays minimal efficiency that progressively decreases with age. In a 60-year-old individual, the proliferative capacity of the adult mammalian heart diminishes to no more than 0.5% [ 1, 2] . Conversely, newborn mammals, including mice and pigs, exhibit robust myocardial regeneration, achieving nearly complete recovery from cardiac injury. However, this regenerative ability sharply diminishes by 7 days after birth [ 3, 4] . Several mechanisms have been identified to promote myocardial regeneration. Li *et al*. [5] reported that metabolic reprogramming enables heart regeneration in adult mice, whereas Feng *et al*. [6] identified the versican protein as a novel target through extracellular matrix analysis. Additionally, Mahmoud *et al*. [7] elucidated the role of the transcription factor Meis1 in regulating cell cycle arrest in cardiomyocytes. Although these studies shed light on inherent myocardial proliferation during the first 7 days after birth, the gradual loss of this proliferative capacity remains insufficiently understood. Notably, it has been clearly reported that cardiomyocytes in suckling mice possess regenerative capacity, which is subsequently lost at postnatal day 7 (P7) and replaced by a fibrotic and apoptotic response leading to cardiac death. In this study, we selected P8 on the basis of the findings of Wang *et al*. [8], who convincingly demonstrated the loss of regenerative capacity in mouse hearts at P8.

Extracellular vesicles (EVs) serve as vital biological carriers, facilitating the transport of diverse cargoes, including lipids, proteins, and various types of RNA, thereby enabling efficient cell-to-cell communication [9]. While much research has focused on EVs obtained from body fluids or cell culture supernatants, the exploration of EVs released by organ tissues remains limited [10]. The heart, a complex organ comprising diverse cell types, such as cardiomyocytes, endothelial cells, fibroblasts, and macrophages, releases EVs containing genetically regulated substances in response to various stresses and stimuli, thereby establishing intercellular homeostasis [ 11, 12] . However, investigations examining alterations in cardiac EVs derived from the heart tissues of mice during the regenerative phase (P1) and the nonregenerative phase (P8) remain scarce. Elucidating the disparities between these distinct phases may provide insights into the loss of proliferative capacity in cardiomyocytes.

Among the diverse cell types present in the heart, macrophages exhibit a remarkable ability to internalize extracellular vesicles (EVs) owing to their distinctive phagocytic capacity [ 13, 14] . However, excessive uptake of EVs by macrophages can impede their effective delivery in EV-based therapies [15]. Therefore, it is reasonable to hypothesize that cardiac macrophages represent the primary cell type directly influenced by cardiac EVs. Furthermore, it has been reported that macrophages derived from the hearts of mice at postnatal day 1 (P1) play a regenerative role in myocardial tissue, yet this function gradually diminishes with increasing postnatal time [ 16, 17] . By investigating alterations in the content of cardiac EVs at P1 and P8, we may elucidate the underlying reasons for this phenomenon.

In this study, we conducted the first isolation and identification of cardiac extracellular vesicles (EVs) derived from heart tissues of P1 and P8 mice, hereafter referred to as P1/P8-EVs. Through differential mRNA cargo analysis, we discovered a significant association of P8-EVs with the immune system. Furthermore, by integrating the results of single-cell sequencing of the hearts of P1/P8 mice, we observed a close correlation between changes in macrophage gene expression and alterations in the mRNA cargo of P1/P8-EVs. Through the integration of transcriptomic expression in macrophages interacting with the myocardium post-cardiac injury, we identified a crucial mRNA, Pak2, within P8-EVs that can induce functional changes in macrophages. Importantly, our findings demonstrate that macrophages internalized by P8-EVs actively suppress myocardial proliferation in neonatal mice and that this inhibitory effect can be reversed by Pak2 inhibitors. Mechanistically, this inhibition is attributed to macrophage activation of the Erk1/2 pathway. Pak2, a serine/threonine protein kinase, plays a pivotal role in various signaling pathways, including cytoskeletal regulation, cell motility, cell cycle progression, apoptosis, and proliferation. Erk, the prototypical MAPK, undergoes phosphorylation to modulate gene expression, thereby promoting growth, differentiation, or mitosis. The phosphorylated and activated form of Erk is referred to as P-Erk. By phosphorylating and activating or inhibiting multiple target proteins, Erk regulates cell growth, differentiation, and death [18]. Subsequent M1 polarization and enhanced phagocytic ability occur following the uptake of P8-EVs by macrophages. Overall, our study reveals a novel mechanism underlying the loss of myocardial proliferation in P8 mice, shedding light on the role of immune cells and cardiac EVs in this process.

## Materials and Methods

### EV isolation

Heart tissue EVs were separated via differential centrifugation. Extraction was performed as follows: left ventricular tissue was cut into small pieces and transferred to a 15-mL centrifuge tube containing 5 mL of type II collagenase (10 mg/mL), and suckling mouse hearts were directly cut, digested with type II collagenase, and incubated for 30 min at 37°C on a shaker with shaking. During the 30-min incubation, the tissue was gently blown up and down with a pasteurized pipette every 10 min to promote myocardial tissue digestion. After digestion, the tissue was centrifuged at 300 *g* for 5 min at 4°C, the supernatant was transferred to a new centrifuge tube and centrifuged at 2000 *g* for 10 min at 4°C, the supernatant was retained and centrifuged at 10,000 *g* for 10 min at 4°C to remove cellular debris, and the supernatant was transferred to an ultracentrifuge tube. The supernatant was transferred to an ultracentrifuge tube, which was subsequently centrifuged at 120,000 *g* for 2 h at 4°C on an Optima XPN-100 ultracentrifuge (Beckman Coulter, Brea, USA), washed once with PBS to obtain EVs, and resuspended in the appropriate amount of PBS.

### Transmission electron microscopy (TEM)

Freshly isolated cardiac EVs were fixed with 2.5% glutaraldehyde fixative. EV suspensions were loaded onto 200 Mesh carbon-coated formvar grids for 5 min and then stained with 2% phosphotungstic acid for 5 min at room temperature. EV samples were examined with a transmission electron microscope (HT7700; Hitachi, Tokyo, Japan).

### Nanoparticle tracking analysis (NTA)

The particle size and concentration of EVs were detected using Zeta View NTA technology, and the process was repeated three times. The analysis was performed as follows: the instrument was automatically calibrated to pass the test before it was used for detection, and the instrument was rinsed so that the number of particles was in the single digits. The exosome sample was dilute with a clean PBS solution to a suitable concentration so that the number of particles displayed in the exosome sample within the instrument’s detection interface was between 50 and 400, preferably around 200. The number of particles displayed at each detection position was confirmed to be within the range before starting the test.

### Western blot analysis

The protein concentration was determined using the bicinchoninic acid (BCA) protein assay kit (Bio-Rad, Hercules, USA). Aliquots of protein samples were separated by sodium dodecyl sulfate-polyacrylamide gel electrophoresis and transferred to polyvinylidene difluoride (PVDF) membranes. The membranes were blocked with 5% skim milk for 1 h at room temperature and then incubated with specific antibodies against CD63 (1:1000 dilution; Abcam, Cambridge, UK), CD81 (1:1000 dilution; Abcam), Alix (1:1000 dilution; Abcam), Erk1/2/p-Erk1/2 (1:1000 dilution; Servicebio, Wuhan, China) and AKT/p-AKT (1:1000 dilution; Servicebio) overnight at 4°C. The next day, the membranes were washed and incubated with a horseradish peroxidase (HRP)-conjugated secondary antibody (1:1000 dilution; Cell Signaling Technology, Danvers, USA). The protein bands were detected with FeiMin™-type ECL luminescent solution (SB-WB004; Sharebio, Shanghai, China) and quantified with an imaging system.

### RNA sequencing

RNA sequencing technology was used to detect the RNA expression profiles of two groups of EVs from different sources: Total RNA from extracellular vesicles was extracted from neonatal murine heart tissues prepared on postnatal day one (P1) and postnatal day eight (P8) using homogenisation with TRIzol (Thermo Fisher Scientific, Waltham, USA). Subsequently, sequencing libraries were constructed using the NEBNext Ultra RNA Library Prep Kit for Illumina (E7530L; NEB, Massachusetts, USA) according to the manufacturer′s instructions.Index codes were then added to individual samples using the NEBNext Ultra RNA Library Prep Kit for Illumina according to the manufacturer’s instructions. The resulting libraries were combined and sequenced on the Illumina platform. Sequencing was performed on the Illumina platform based on the optimal library concentration and data volume required. Sequencing was performed on the Illumina platform based on the optimal library concentration and data volume required. Clustering analysis, GO analysis, and KEGG pathway analysis were performed to predict the target genes and target pathways related to the myocardial proliferation mechanism.

### Data collection

Single-cell data from mice at postnatal days 1 and 8 were obtained from the Gene Expression Omnibus (GEO) database dataset GSE153480, and single-cell RNA sequencing data were downloaded from the ArrayExpress ( https://www.ebi.ac.uk/arrayexpress/) database for post-infarction mice.

### Single-cell analysis

All analyses in the study were performed using R software (version 4.1.3), and the scRNA-seq data were analyzed using the Seurat package (version 4.2). Low-quality cells with fewer than 300 or more than 7500 expressed genes or with more than 25% unique molecular identifiers (UMIs) in the mitochondrial genome were removed. The mitochondrial, ribosomal, and hemoglobin genes were then removed from the dataset. Finally, the genes to be analyzed were obtained.

LogNormalize was used to normalize the genes in the dataset. The top 2000 highly variable genes were identified via the FindVariableFeatures function in the Seurat package with default parameters. Principal component analysis (PCA) was performed on the highly variable genes after Z score normalization. Uniform manifold approximation and projection (UMAP) was performed to downscale the first 16 significant principal components (pc). Clusters were identified via the FindClusters function (resolution = 1.2). The marker genes for each cluster were identified as having greater than 2-fold variation via the Seurat FindMarkers function. Known cell types were marked with the marker gene CellMarker 2.0 [19].

The workflow of the hdWCNA was executed via the R package “hdWGCNA”. Briefly, the hdWCNA pipeline consists of the following steps: (1) data preprocessing; (2) gene network construction; (3) module identification; and (4) module preservation analysis. In the first step, the gene expression data are preprocessed to remove noise and batch effects. In the second step, gene co-expression networks were constructed on the basis of pairwise correlations between genes. In the third step, modules or gene clusters of highly correlated genes were identified, and the characteristic genes of the modules were calculated. In the fourth step, module preservation analysis was performed to assess the robustness of the identified modules.

### Immunofluorescence staining

The macrophages were cocultured with the lipophilic dye 1,1′-dioctadecyl-3,3,3′,3′-tetramethyl polycarbocyanine perchlorate (DiI, 10 μM; Beyotime, Shanghai, China) labelled with EVs for 24 h. The cells were rinsed with PBS and then fixed with 4% (w/v) paraformaldehyde for 15 min. Ghost pen cyclic peptide (Yeasen Biotechnology, Shanghai, China) was used to stain the macrophage cytoskeleton, and DAPI (Yeasen Biotechnology) was used for nuclear staining.

After EV-treated macrophages were cocultured with cardiomyocytes for 24 h, cardiomyocytes were labelled with antibody against CTNT (1:200 dilution; Abclonal, Wuhan, China), and the proliferation of cardiomyocytes was identified by BrdU (1:200 dilution; Abclonal) and Ki67 (1:200 dilution; Abclonal) assays.

### Flow cytometry

The RAW264.7 macrophages were treated with P1-EVs or P8-EVs and then cultured in 1640 medium (supplemented with 10% fetal bovine serum (FBS) and 1% penicillin-streptomycin) for 48 h at 37°C and 5% CO _2_. The cells were collected, rinsed with PBS, processed with an antibody against CD86 (1:200 dilution; Proteintech, Wuhan, China), and centrifuged. The PBS-treated cells were subsequently collected, and the polarization of the macrophages was detected with a FACSCanto II flow cytometer (BD Bioscience, Franklin Lakes, USA).

### Animals

C57BL/6J wild-type mice (male, 6‒8 weeks old) were purchased from Shanghai Jiesijie Laboratory Animal Co. (Shanghai, China). The mice were housed at specific pathogen-free (SPF) room temperature (23‒24°C), humidity (55% ± 5%), and light (12/12-h height/dark cycles), and all the mice were not exposed to food or water. The experimental protocols were approved by the Animal Protection and Utilization Committee of Tongji University (No. TJBB06123101). Computer-generated random numbers were used for random grouping in this study.

### MI model

Permanent left anterior descending ligation or sham surgery was performed on the experimental animals. Briefly, the mice were anaesthetized via intraperitoneal injections of anesthetics. A small skin incision (1.2 cm) was subsequently made in the left chest, and the LAD was permanently ligated using a 6-0 silk suture. The suture was passed 2–3 mm below the tip of the left auricle. Mice that did not survive 24 h after surgery were excluded from the analysis. Sham-operated animals underwent the same procedure but without ligation of the coronary artery. At the indicated time points, the mice were euthanized by CO2/cervical dislocation, and the tissues were subsequently collected for analysis.

### Isolation of cardiomyocytes and treatments

Neonatal murine hearts were dissected and carefully cut into small pieces with fine scissors and then treated with type II collagenase (1.5 mg/mL; Worthington Biochemical, Lakewood, USA) or elastase (0.25 mg/mL; Worthington Biochemical) for 1 h at 37°C. The tissue was digested and then centrifuged at 210 *g* for 5 min. After digestion, the tissues were passed through a 70-μm cell strainer and then centrifuged at 210 *g* for 5 min, and the resulting precipitate was subsequently lysed in erythrocyte solution, centrifuged again, washed with PBS and spread on a plate. Cardiomyocytes were cultured in DMEM (Gibco, Carlsbad, USA) supplemented with 10% FBS and 1% penicillin/streptomycin at 37°C with 5% CO _2_. FRAX486 is a Pak inhibitor purchased from MCE (Monmouth Junction, USA). The IC _50_ values for Pak1, Pak2, and Pak3 were 14, 33, and 39 nM, respectively. On the basis of the exploratory results of Wang *et al*. [20], we treated the cells with 5 μM FRAX486 for 24 h.

To investigate how macrophages that phagocytose extracellular vesicles affect cardiomyocytes. We simulated *in vitro* the interaction between macrophages and cardiomyocytes in vivo, we cultured macrophages on transwell chambers with a pore size of 0.4 μm under the following conditions: DMEM (Gibco, Carlsbad, USA) with 10% fetal bovine serum and 1% penicillin/streptomycin, 37°C, and 5% CO _2_. Then equal amounts (2 μg) of P1-EVs, P8-EVs, PBS and P8-EVs + Pak2 inhibitor were added to the macrophages, and then the transwell chambers were placed on the neonatal rat cardiomyocytes extracted above for co-culture. The culture conditions of cardiomyocytes were as described previously. We then examined the proliferation of each group of cardiomyocytes by immunofluorescence.

### Statistical analysis

Data are expressed as the mean ± standard error of the mean (SEM). Student’s *t* test was used to compare two groups. Multiple group comparisons were made via one-way analysis of variance (ANOVA) followed by the Student-Newman-Keuls post hoc test or nonparametric Mann-Whitney U test. All the data were analyzed via GraphPad Prism 7.0 (GraphPad Software, San Diego, USA), and *P*  < 0.05 was considered to indicate a statistically significant difference.

## Results

### Isolation and identification of cardiac EVs from mice at P1/P8

**Figure 1:**
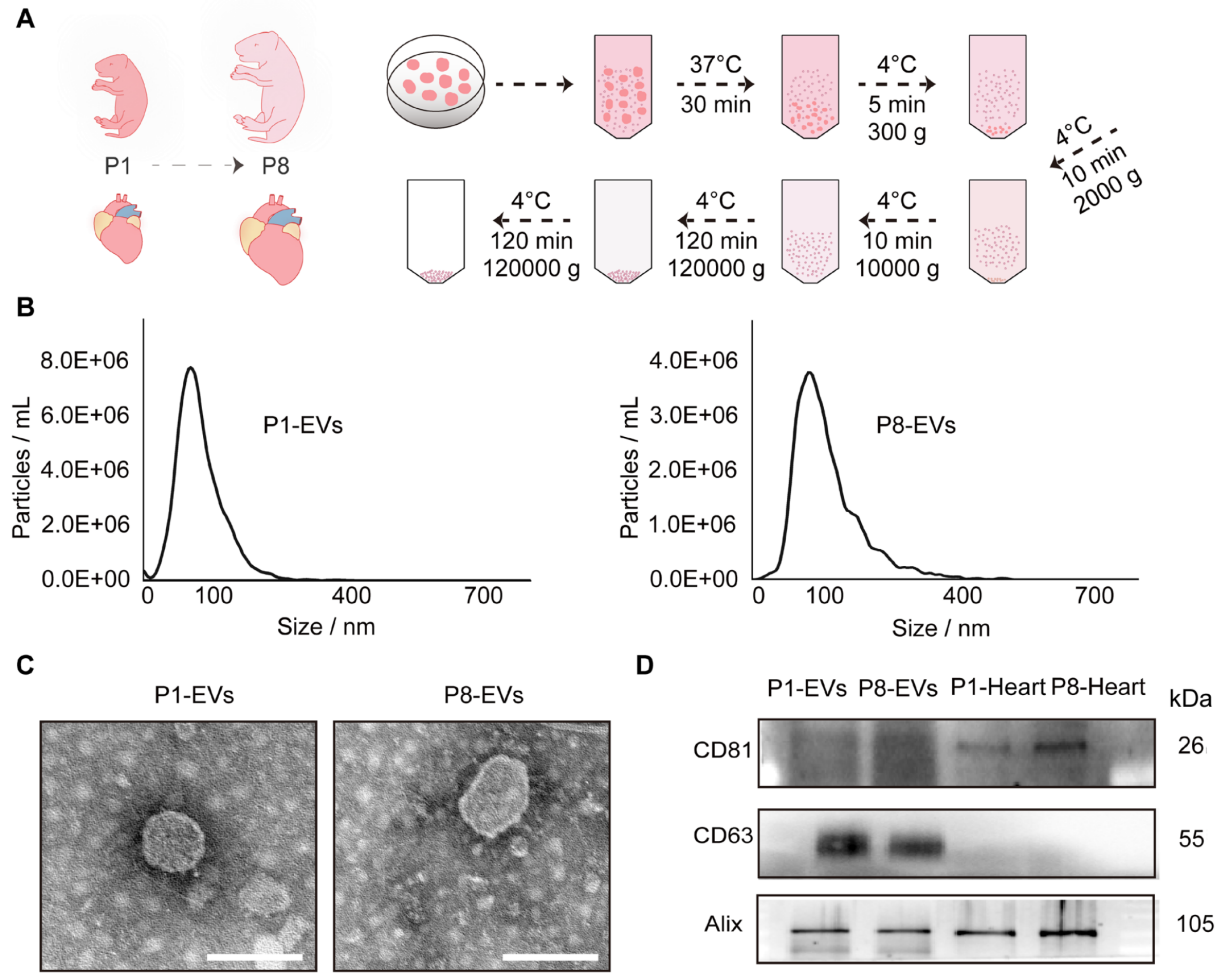
Figure 1 Isolation and identification of P1/P8-EVs (A) Cardiac EV isolation process. (B) Size distribution analysis of P1/P8-EVs. (C) Representative TEM images of P1/P8-EVs. Scale bar: 100 nm. (D) Representative western blots of three typical markers (CD81, CD63 and Alix) in P1/P8-EVs and P1/P8-hearts.

To obtain cardiac EVs, heart tissues from P1 and P8 mice were subjected to shear and collagenase digestion, followed by differential ultracentrifugation and washing steps ( Figure 1A). Nanoparticle tracking analysis (NTA) revealed that the diameters of the EVs obtained from both groups predominantly ranged from 50–150 nm ( Figure 1B), which is consistent with the typical size range of EVs (30–200 nm). Moreover, transmission electron microscopy (TEM) images revealed the characteristic round or cup-shaped morphology of the EVs ( Figure 1C). Additionally, western blot analysis results confirmed the presence of classical protein markers, including CD81, CD63, and Alix ( Figure 1D). Collectively, these findings provide conclusive evidence of the successful isolation of P1 and P8 cardiac EVs.

### Lack of impact of P1/P8-EVs on neonatal mouse cardiomyocyte proliferation and post-infarction myocardial repair

**Figure 2:**
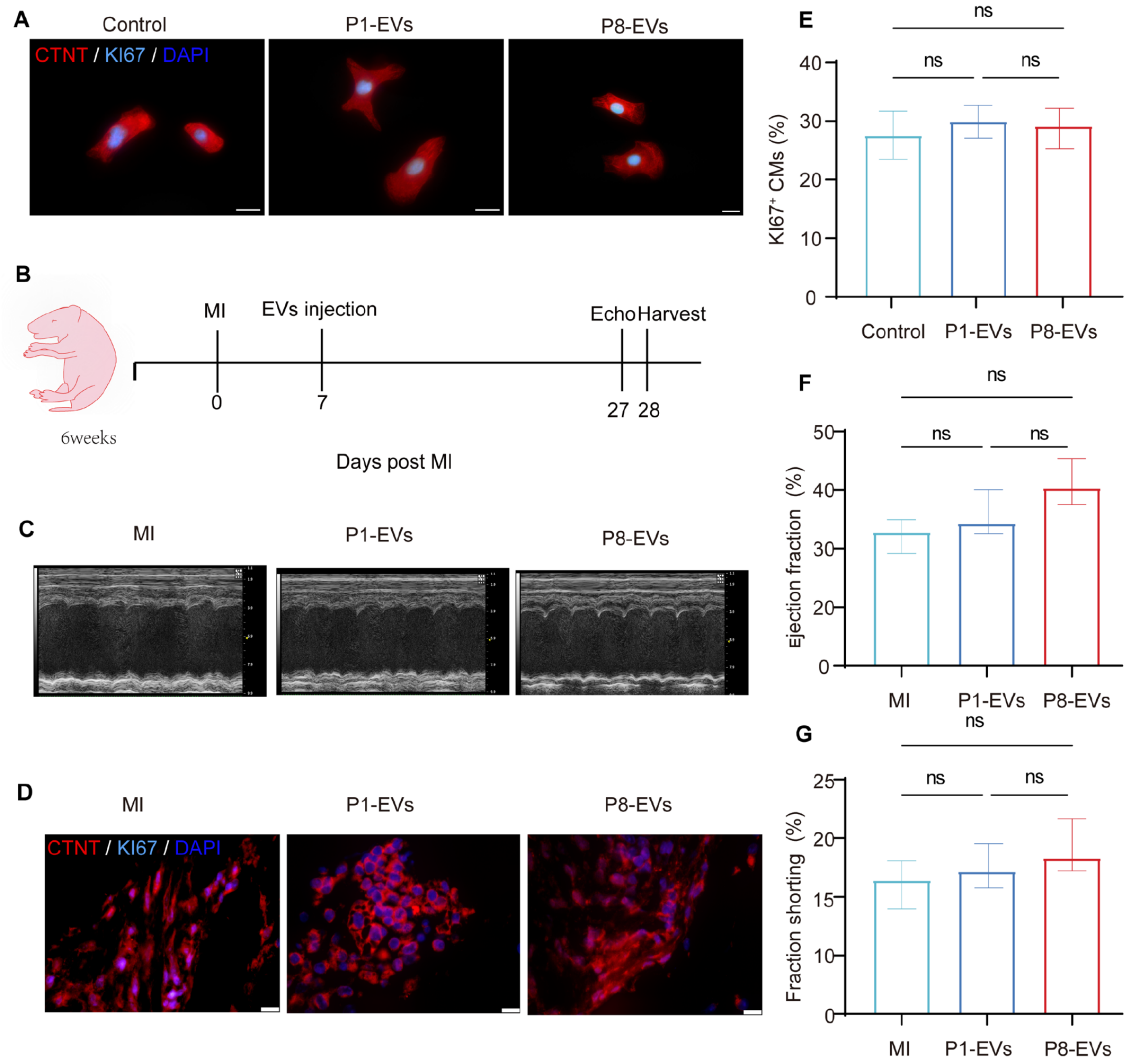
Figure 2 Effects of P1/p8-EVs on neonatal mouse CM proliferation and post-infarction myocardium (A) CTNT (red), Ki67 (cyan), and DAPI (blue) staining of cardiomyocytes co-culture with different extracellular vesicles (EVs). Thirty-seven Ki67-positive cardiomyocytes in the control group; 29 Ki67-positive cells in 105 cardiomyocytes in the P1 group; 35 Ki67-positive cells in 126 cardiomyocytes in the P8 group. Scale bar: 20 μm. (B) Experimental program for EV treatment of mice with myocardial infarction. (C) Representative M-mode echocardiograms. Echocardiographic data represent biologically independent mice in each condition, distributed among three cohorts (n = 5). (D) CTNT (red), KI67 (cyan), and DAPI (blue) staining of proliferation in EV-treated and untreated cardiomyocytes. Scale bar: 20 μm. (E) Statistics on the proliferation of cells in (A) (n = 3). (F,G) Statistics on (C) Cardiac Ejection Fraction and Shortening Fraction of Echocardiography (n = 5).

Following 48 h of direct co-culture of tissue exosomes with cardiomyocytes, Ki67-stained images revealed no discernible effect on cardiomyocyte proliferation upon direct treatment with P1-EVs or P8-EVs ( Figure 2A,E). Additionally, the entire experimental protocol involving surgical treatment of the mice, subsequent administration of P1-EVs/P8-EVs, and echocardiography examination four weeks later is illustrated in Figure 2B. In line with the *in vitro* findings, no significant effect on the ejection fraction or left ventricular short-axis shortening rate [ 21, 22] was observed in infarcted mice following intramyocardial injection of P1/P8-EVs (Figure 2C, 2F,G). Moreover, we collected heart sections from each group for staining, and the staining results revealed no proliferation, indicating that EVs do not directly affect cardiomyocyte proliferation ( Figure 2D).

### Analysis of mRNA cargo enclosed within P1/P8-EVs

**Figure 3:**
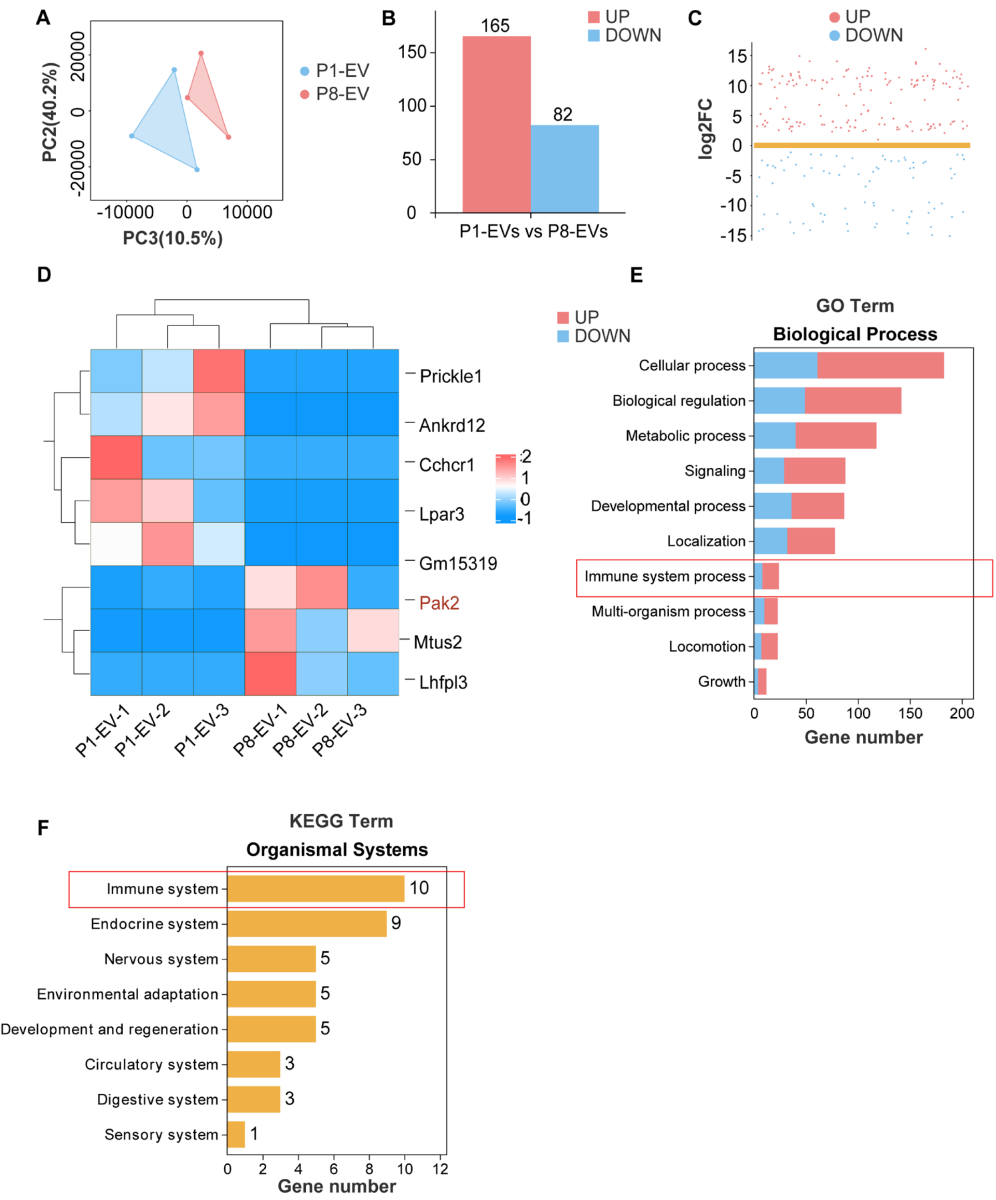
Figure 3 Analysis of mRNA cargo encapsulated in P1/P8-EVs (A) PCA plot of gene expression in P1-EVs and P8-EVs. (B,C) Bar plots and scatter plots illustrating genes differentially expressed between P1-EVs and P8-EVs (log2FC ≥ 1.5 and P < 0.05). (D) Heatmap showing the selected upregulated and downregulated genes. (E) Pathways associated with the biological process of GO enrichment analysis for upregulated DEGs in P8-EVs. (F) Number of upregulated DEGs in P8-EVs associated with pathways in various systems according to KEGG enrichment analysis.

After the isolation of P1/P8 EVs, we performed mRNA sequencing to investigate the disparities in their cargo. A total of 2975 mRNAs were identified within the P1/P8 EVs, and principal component analysis (PCA) demonstrated robust reproducibility across batches, along with significant distinctions between the groups ( Figure 3A). Among these genes, we identified 247 differentially expressed genes (DEGs), with 165 genes upregulated in P8-EVs compared with P1-EVs and 82 genes downregulated ( Figure 3B,C). In addition, we also displayed partial differential genes, as shown in Figure 3D. To gain further insight, the DEGs were subjected to GO enrichment analysis, which revealed significant enrichment in biological processes associated with the immune system ( Figure 3E). Additionally, KEGG enrichment analysis revealed that the enriched pathways are related primarily to the immune system ( Figure 3F). Collectively, these findings suggest that the modulation of immune-related responses by cardiac EVs from P1 to P8 may play a crucial role in the loss of myocardial proliferation.

### Macrophages are the primary target cells of P1/P8-EVs

**Figure 4:**
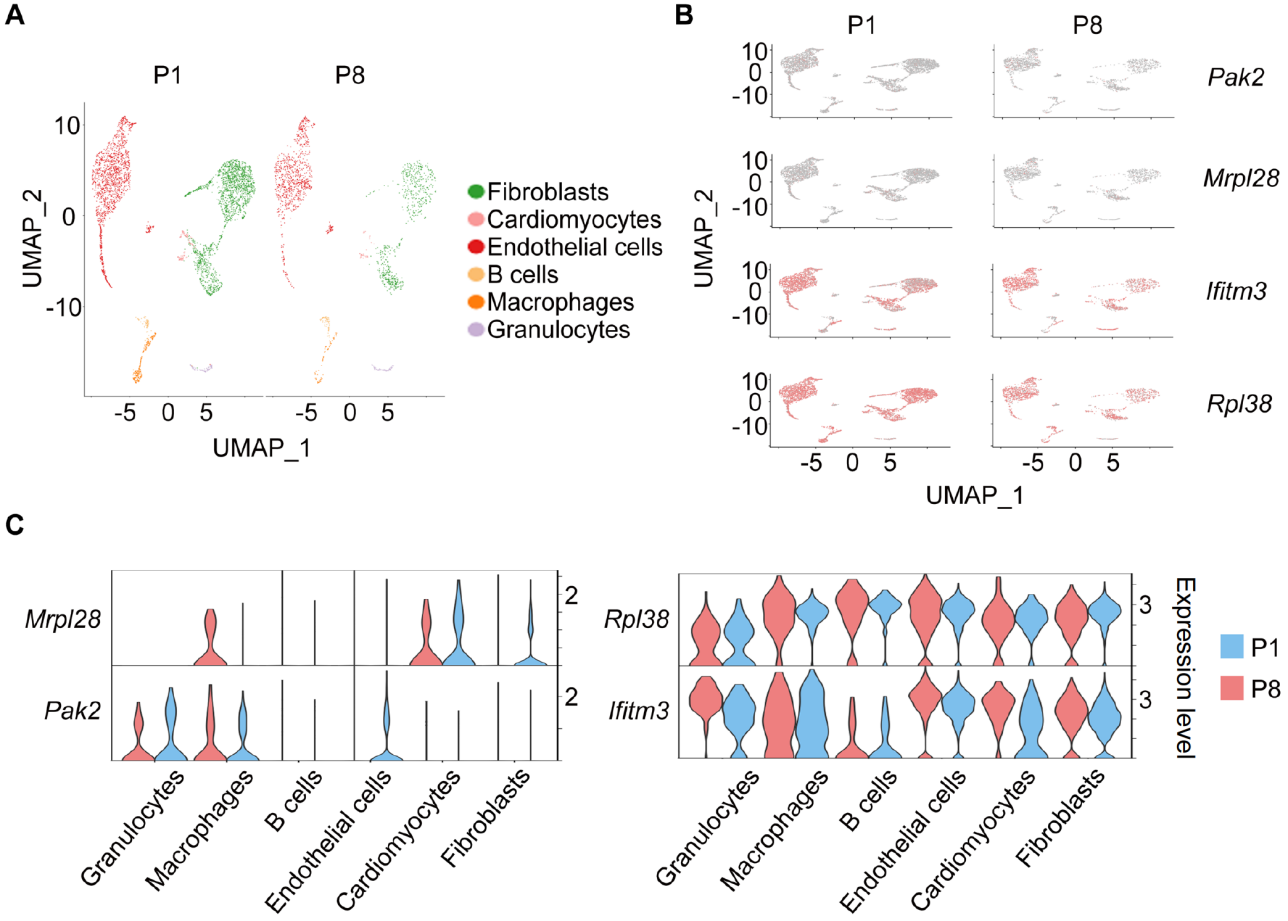
Figure 4 Single-cell analysis of P1/P8 hearts revealed the primary target cells for P1/P8-EVs (A) UMAP plots of single cardiac cells at P1/P8. (B) Distribution of some mRNAs upregulated in P8-EVs in cell clusters of P1/P8 hearts (Pak2, Mrpl28, Ifitm3, and Rpl38). (C) Violin plots of the expressions of the above mRNAs in different cell clusters.

Given the intricate cellular composition of the heart, determining the specific target cells and their functional roles on the basis solely of the mRNA cargo within exosomes is challenging, as they are co-secreted by multiple cell types. To address this, we downloaded the publicly available dataset GSE153480 from the GEO database, which provides comprehensive sequencing of cardiac single cells obtained from mice on postnatal days 1 and 8, enabling a more informed assessment. On the basis of previous reports, six major cell populations, namely, fibroblasts, cardiomyocytes, endothelial cells, B cells, macrophages, and granulocytes, were identified after unsupervised clustering and dimensionality reduction ( Figure 4A). Furthermore, we observed significant upregulation of certain mRNAs, such as Pak2, Mrpl28, Ifitm3, and Rpl38, in P8-EVs compared with P1-EVs, which are specifically associated with distinct clusters ( Figure 4B). Notably, among these cell populations, macrophages exhibited a pronounced trend consistent with the upregulation of P8-EV mRNA, suggesting that EVs secreted by various cardiac cell types are predominantly internalized by macrophages upon release. This phenomenon likely contributes to the increase in Pak2 expression, specifically in macrophages, in P8 mice ( Figure 4C). These results strongly indicate that alterations in the mRNA cargo of P8-EVs, compared with those of P1-EVs, are closely linked to changes in macrophages, thus offering potential insights into their role in myocardial proliferation.

### *Pak2*, enriched in P8-EVs, is a crucial gene involved in regulating macrophage functions during myocardial injury repair

To elucidate the impact of EV changes induced by macrophages on the decline in myocardial proliferation, it is imperative to identify mRNAs capable of activating macrophages and influencing cardiomyocytes. Following myocardial infarction, macrophage function is significantly altered, thereby amplifying myocardial injury. The transcriptome changes in macrophages may provide valuable insights into their interaction with cardiomyocytes.

**Figure 5:**
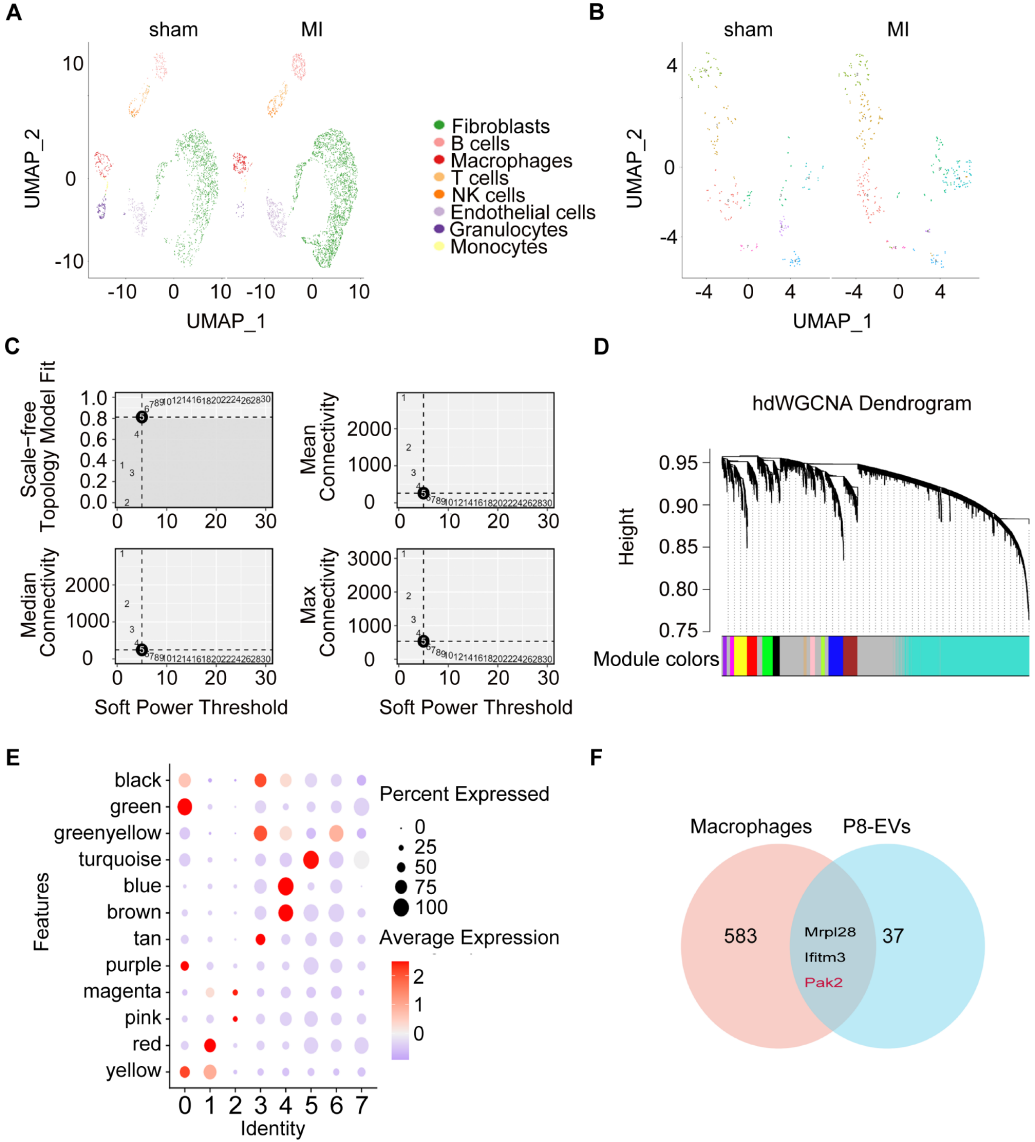
Figure 5 Integration analysis reveals that pak2 is a key gene that regulates macrophage function during myocardial injury repair in P8-EVs (A) UMAP plots of single cardiac cells from sham and myocardial infarction hearts. (B) UMAP plots of macrophage populations from the sham and myocardial infarction groups. (C–E) HdWGCNA of genes from macrophage clusters associated with myocardial infarction. (F) Wayne plots of genes identified by single-cell analysis with highly expressed mRNAs in P8-EVs.

We obtained the MTAB-9816 single-cell dataset from the Array Express database, which examines cardiac single cells after myocardial infarction ( Figure 5A). From this dataset, we extracted macrophage clusters. Interestingly, not all macrophage clusters exhibited universal upregulation after infarction; rather, clusters 0, 1, and 4 showed notable upregulation ( Figure 5B). To characterize each cluster, we performed hierarchical dynamic WGCNA (hdWGCNA) on macrophages. A power of 5 was chosen to construct a scale-free network, resulting in 12 gene modules ( Figure 5C,D). Among these modules, the green, blue, brown, and red modules are particularly associated with post-infarction changes in macrophages, as evidenced by the fractional dot plots ( Figure 5E). By intersecting the genes within these modules with the highly expressed mRNAs in P8-EVs, we identified three key mRNAs, Mrpl28, Ifitm3, and Pak2 ( Figure 5F), as the most promising targets involved in activating macrophages and influencing cardiomyocytes.

### P8-EVs inhibit neonatal cardiomyocyte proliferation by mediating Pak2-dependent macrophage paracrine signaling

**Figure 6:**
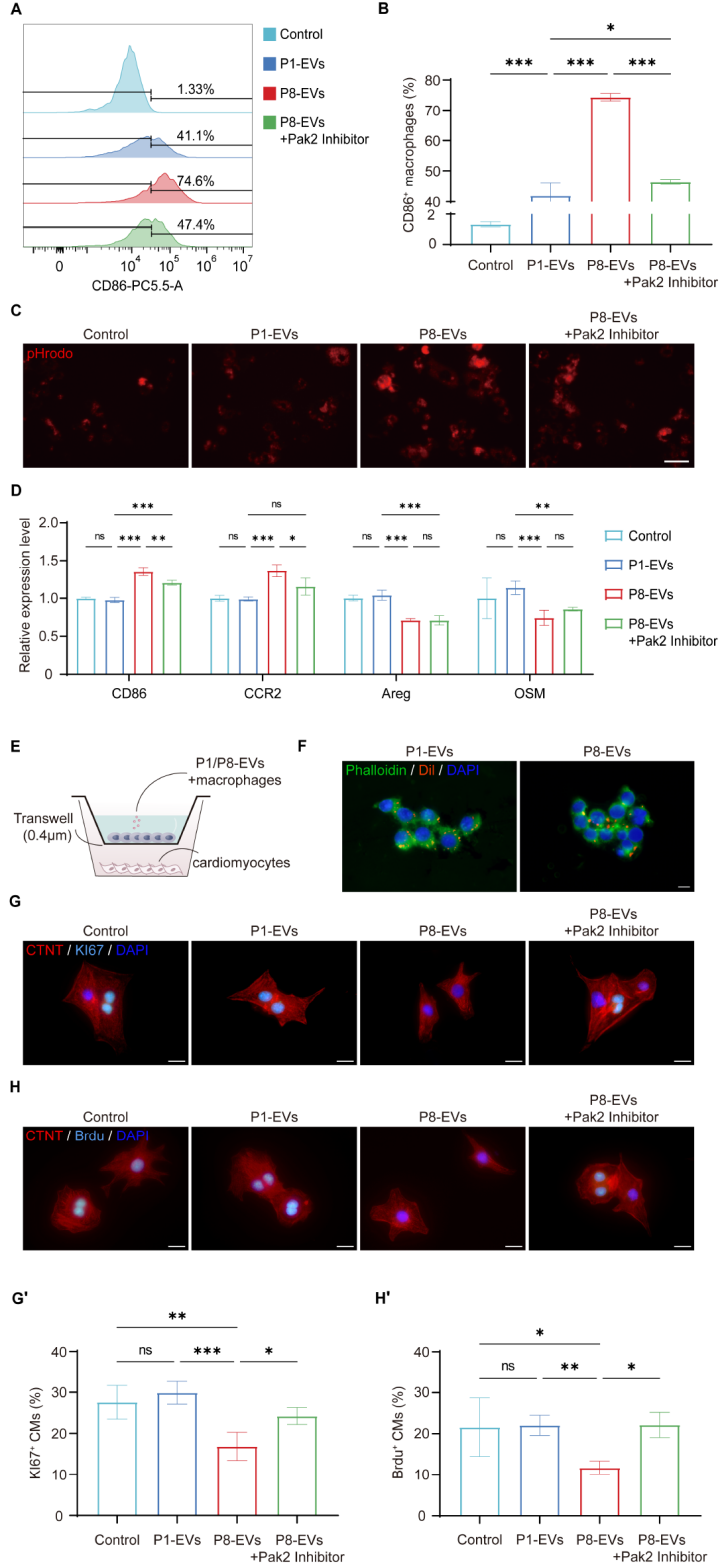
Figure 6 P8-EVs inhibit cardiomyocytes proliferation by modulating the macrophage phenotype (A) Flow cytometry analysis of macrophages internalizing P1/P8-EVs expressing the M1 macrophage polarization marker CD86. (B) The proportion of CD86-positive macrophages (n = 3). (C) The phagocytic function of macrophages was assayed with pHrodo dye (red). Scale bar: 50 μm. (D) mRNA expression levels of CD86, CCR2, Areg and OSM (n = 3). (E) Coculture system of P1/P8-EVs, macrophages and cardiomyocytes. (F) Phalloidin (green) and DAPI (blue) staining of macrophages internalized Dil-labelled (red) P1/P8-EVs. Scale bar: 10 μm. (G) CTNT (red), KI67 (cyan) and DAPI (blue) staining of cardiomyocytes co-culture with differently treated macrophages. There were 36 Ki67-positive cells in 137 cardiomyocytes in the control group; 36 Ki67-positive cells in 129 cardiomyocytes in the P1 group; 17 Ki67-positive cells in 119 cardiomyocytes in the P8 group; and 31 Ki67-positive cells in 120 cardiomyocytes in the P8+Pak2 inhibitor group. Scale bar: 20 μm. (H) Immunofluorescence staining of CTNT (red), BrdU (cyan) and DAPI (blue) in cardiomyocytes co-culture with differently treated macrophages. 46 Ki67-positive cells out of 191 cardiomyocytes in the control group; 35 Ki67-positive cells out of 147 cardiomyocytes in the P1 group; 13 Ki67-positive cells out of 100 cardiomyocytes in the P8 group; 21 Ki67-positive cells out of 95 cardiomyocytes in the P8 + Pak2 inhibitor group. Scale bar: 20 μm. (G′-H′) Statistical analysis of the above data (n = 3). *P < 0.05, **P < 0.01.

On the basis of single-cell analysis demonstrating that macrophages predominantly phagocytose exosomes in tissues, we further investigated macrophages. Flow cytometry analysis revealed that macrophages internalized by P8-EVs were predominantly polarized toward the M1 phenotype. However, treatment with Pak2 inhibitors alleviated this phenotype and restored polarization levels similar to those observed with internalized P1-EVs ( Figure 6A,B). Additionally, we assessed the phagocytic function of macrophages via pHrodo dye, which significantly increased phagocytic activity following treatment with P8-EVs. In contrast, P1-EVs and Pak2-inhibited P8-EVs did not exhibit such effects ( Figure 6C). Analysis of gene expression in macrophages revealed a consistent trend with the flow cytometry analysis. The expression of a marker of cardiac resident macrophages, CCR2, decreased after P8-EVs were internalized, with a slight upwards trend observed after the addition of a Pak2 inhibitor. Both the macrophage-secreted pro-myocardial proliferative factors Areg and OSM exhibited decreased expression in the presence of P8-EVs, which could not be restored even with Pak2 inhibitors ( Figure 6D). Overall, P8-EVs promoted M1 polarization and enhanced phagocytic function in macrophages, while the expressions of tissue-resident markers and proliferation factors decreased. The Pak2 inhibitor partially reversed M1 polarization and phagocytic function but had no effect on the latter two factors.

Next, we sought to investigate how macrophages that engulf extracellular vesicles affect cardiomyocytes. To simulate the *in vivo* interaction between macrophages and cardiomyocytes, we cultured macrophages on transwell inserts with a pore size of 0.4 μm, while cardiomyocytes from P1 mice were placed in the lower chamber. P1/P8-EVs were introduced into the culture system ( Figure 6E). Considering that Pak2 mRNA is a potential key target, we co-administered a Pak2 inhibitor and P8-EVs as an additional experimental group to observe phenotypic changes. Immunofluorescence staining confirmed the successful uptake of P1/P8-EVs by macrophages ( Figure 6F). Ki67 staining images revealed that while untreated macrophages and those internalized by P1-EVs had no effect on cardiomyocyte proliferation, macrophages internalized by P8-EVs significantly suppressed cardiomyocyte proliferation. Importantly, this inhibitory effect was reversed upon the addition of the Pak2 inhibitor ( Figure 6G,G′). To further confirm these findings, we added BrdU to the culture medium as a proliferation marker and performed staining after co-culture. The observed alterations in proliferative capacity were consistent with the Ki67 staining results ( Figure 6H,H′). These results suggest that macrophages internalized by P8-EVs exert an inhibitory effect on cardiomyocyte proliferation, which is mediated by Pak2 mRNA.

### P8-EVs activate Erk1/2 signaling pathways in macrophages via Pak2

**Figure 7:**
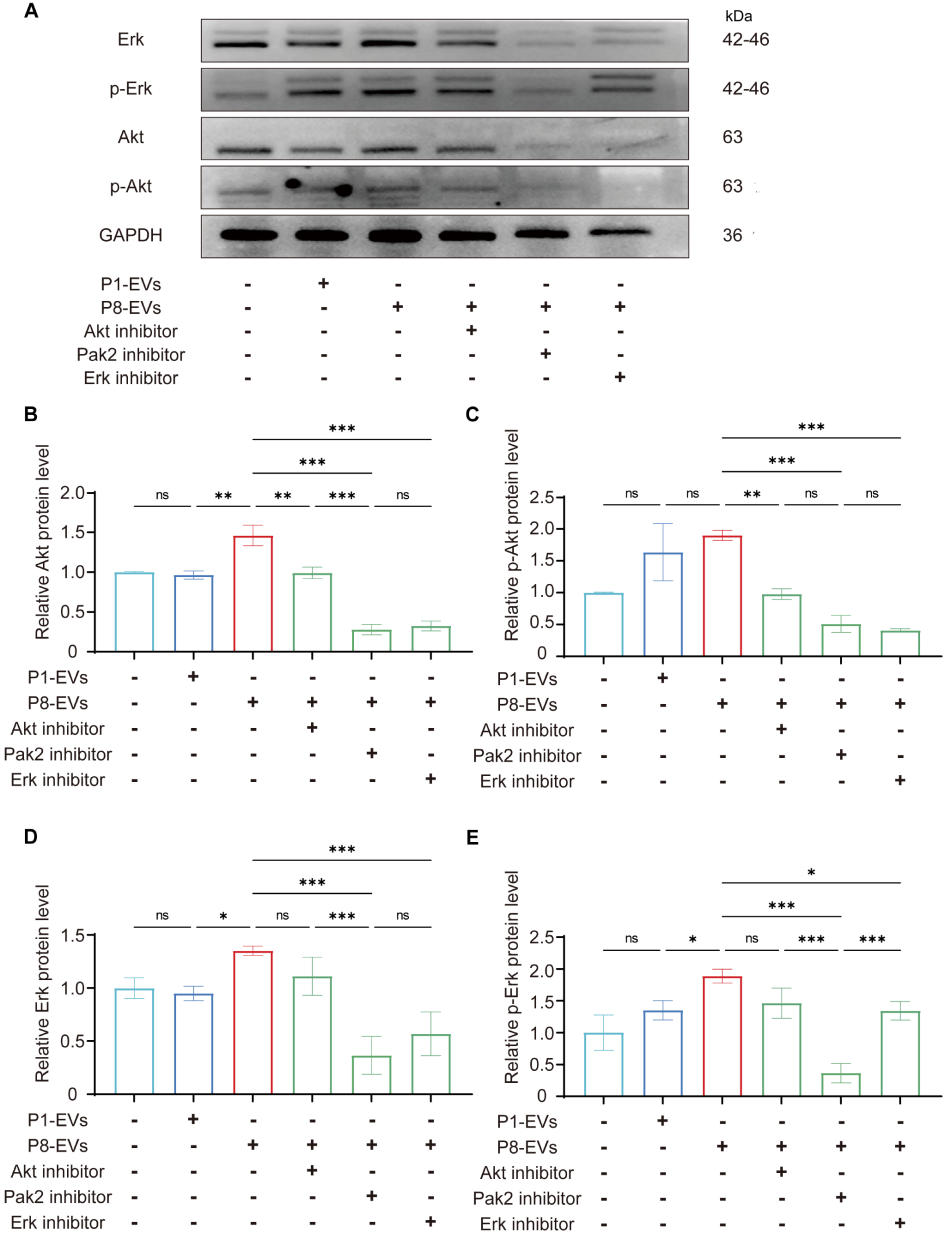
Figure 7 P8-EVs activate the Erk1/2 pathway in macrophages via Pak2 (A) Representative western blots of Erk1/2, p-Erk1/2, Akt, p-Akt and GAPDH in macrophages subjected to different treatments. (B–E) Quantitative analysis of the band intensities in (A). *P < 0.05, **P < 0.01, ***P < 0.001.

To investigate the downstream pathways involved in the internalization of P8-EVs by macrophages, we performed western blot analysis of the classical Akt and Erk1/2 signaling pathways ( Figure 7A). The images and statistical results indicated limited alterations in phosphorylated Akt protein levels when P1-EVs and P8-EVs were compared, despite higher total Akt protein expression in macrophages internalizing P8-EVs than in those internalizing P1-EVs ( Figure 7B,C). In contrast, the Erk1/2 pathway was significantly activated following treatment with P8-EVs, as evidenced by increased phosphorylation of Erk1/2. Notably, the addition of Pak2 inhibitors blocked this process to a greater extent than Erk1/2 inhibitors alone did ( Figure 7D,E), suggesting that Pak2 acts upstream of the Erk1/2 signaling pathway. These results suggest that P8-EVs deliver Pak2 into macrophages, thereby activating the Erk1/2 pathway and resulting in significant M1 polarization and enhanced phagocytic function. Ultimately, these processes impact myocardial proliferation.

## Discussion

The fundamental cause of heart failure in adult mammals is the limited regenerative capacity of cardiomyocytes following injury [23]. Therefore, myocardial proliferation in neonatal mammals [24] is crucial. In this study, we investigated the role of EVs and established an effective method for isolating cardiac EVs from the heart tissues of P1/P8 mice. While EVs are known to carry diverse miRNAs, mRNA, proteins, etc., we focused on the mRNA cargo, which is also abundant in EVs [25].

Surprisingly, we found that the highly expressed mRNAs in P8-EVs, compared with those in P1-EVs, were significantly enriched in pathways related to immunity. This finding coincides with recent studies highlighting the impact of immune cells on myocardial proliferation [ 26, 27] . However, most of the current research focuses on how early immune responses promote myocardial proliferation [ 28, 29] , and reports on the role of immune responses in the decline or loss of regenerative capacity are limited. To address this knowledge gap, we performed an integrative analysis of the highly expressed mRNAs in P8-EVs and the highly expressed genes in P8 cardiac cells using single-cell sequencing. The results revealed a significant overlap between the changes in the transcriptome of macrophages from P1 to P8 and the alterations in the mRNA cargo of cardiac EVs. Previous studies have reported two populations of macrophages in the heart: self-renewing resident macrophages and macrophages derived from monocytes [30]. The former are believed to proliferate and accumulate at the injury site to mediate cardiac regeneration [31]. However, as the heart matures, the proportion of the latter population gradually increases [32], which may explain the changes observed in macrophage clusters during single-cell analysis from P1 to P8 and their impact on the cargo of cardiac EVs.

In the context of adult myocardial infarction, resident macrophages are quickly depleted and replaced by macrophages derived from monocytes [33]. Understanding how macrophages derived from monocytes interact with cardiomyocytes provides unique insights into the changes in the proportions and functions of the two macrophage populations from P1 to P8. Therefore, we identified key genes that may simultaneously regulate both macrophages and cardiomyocytes by intersecting the changes in the transcriptome of macrophages after myocardial infarction with the highly expressed mRNAs in P8-EVs.

Among these key genes, Pak2 has been studied in cardiac research [34]. Previous reports have shown that Pak2 can promote a protective endoplasmic reticulum stress response in cardiomyocytes, improve cardiac function, and reduce cell apoptosis in adult mice [35]. However, its role in myocardial proliferation remains unknown. In our co-culture system of cardiac EVs, macrophages, and cardiomyocytes, we observed that macrophages regulated by P8-EVs inhibited cardiomyocyte proliferation, which was partially reversed by the addition of a Pak2 inhibitor. Notably, enhancing endoplasmic reticulum function and inhibiting proliferation are not contradictory and may be categorized as functions that promote cell maturation [36]. The specific phenotypes of Pak2 in relation to these two functions warrant further investigation.

The impact of macrophage polarization on cardiac regeneration remains inconclusive, partly because of the complexity of M1 and M2 polarization and the existence of multiple macrophage subtypes in different microenvironments [37]. Furthermore, we did not inhibit EV secretion to elucidate the effects of macrophages on cardiomyocytes. In this study, we found that macrophages that internalized P8-EVs differentiated toward M1 polarization and demonstrated enhanced phagocytic function. However, further studies are needed to explore the direct relationship between these functional phenotypes and the inhibition of proliferation. Additionally, *in vivo* tests of cardiomyocyte proliferation are essential for identifying regenerative targets and should be considered in future investigations.
